# P-2176. Descriptive Epidemiology of Mpox and Varicella-Zoster Virus (VZV) Co-Infection, January 2022 to March 2025 in Imo State, Southeastern Nigeria

**DOI:** 10.1093/ofid/ofaf695.2339

**Published:** 2026-01-11

**Authors:** Hyacinth Egbuna, Leonard Ihedioha, Evangeline Oparaocha, Ugonma Dozie, Chimezie Iwuala

**Affiliations:** Ministry of Health, Owerri, Imo State, Nigeria, Owerri, Imo, Nigeria; Ministry of Health, Owerri, Imo State, Nigeria, Owerri, Imo, Nigeria; Federal University of Technology Owerri, Imo State, Nigeria, Owerri, Imo, Nigeria; Federal University of Technology Owerri, Imo State, Nigeria, Owerri, Imo, Nigeria; Federal University of Technology Owerri, Imo State, Nigeria, Owerri, Imo, Nigeria

## Abstract

**Background:**

Mpox is a zoonotic disease that is endemic to Nigeria and is caused by the Mpox virus, a member of the orthopoxvirus family. In humans, this virus can produce a severe illness that may resemble chickenpox or smallpox, and it frequently results in co-infections with varicella-zoster viruses (VZV). The first occurrence in Imo State was reported in 2018 and resulted in death. Beginning on January 6, 2022, a rising number of instances signaled the beginning of the outbreak. We looked into the severity of the co-infections of Mpox and varicella-zoster (VZV) outbreaks in the state.

**Methods:**

Using the accepted standard case definition for Mpox, which is defined as a person with an acute illness who has a fever >38.3 °C, a severe headache, lymphadenopathy, back pain, and rashes that spread to every part of the body, including the soles of the feet and palms of the hands. In order to create a line list, we gathered sociodemographic and clinical information. For laboratory validation utilizing reverse transcription polymerase chain reaction (RT-PCR), we collected swabs and blood samples. We computed ratios and rates.

**Results:**

357 suspected cases were found and investigated from January 2022 to March 2025, there were 56 laboratory-confirmed cases of Mpox-VZV coinfection with 2 deaths and the highest proportion of cases being reported from Owerri Municipal LGA (23.8%), males (69.0%), aged 0-5 years (21.4%), and family members as contacts (50.3%). With a case fatality rate of 3.6% and a positivity rate of 15.7%. Fever, palm, foot, and cheek rashes, as well as pustules, were present in all 56 patients.

**Conclusion:**

During the response, we enhanced surveillance for active case search, which helped to identify more cases. In order to encourage early diagnosis and case treatment, there is a need to increase awareness of the condition and its risk factors for high levels of index suspicion. In Nigeria, the introduction of the Mpox and varicella-zoster viruses (VZV) infection vaccines is needed, especially for children under 5 years children because they are mostly infected.

**Disclosures:**

All Authors: No reported disclosures

